# First Autologous Cell Therapy of Cerebral Palsy Caused by Hypoxic-Ischemic Brain Damage in a Child after Cardiac Arrest—Individual Treatment with Cord Blood

**DOI:** 10.1155/2013/951827

**Published:** 2013-05-15

**Authors:** A. Jensen, E. Hamelmann

**Affiliations:** ^1^Campus Clinic Gynecology, Ruhr-University Bochum, Universitätsstrasse 140, 44799 Bochum, Germany; ^2^Department of Pediatrics, Ruhr-University Bochum, St. Josef-Hospital, Alexandrinenstrasse 5, 44791 Bochum, Germany

## Abstract

Each year, thousands of children incur brain damage that results in lifelong sequelae. Therefore, based on experimental evidence, we explored the therapeutic potential of human cord blood, known to contain stem cells, to examine the functional neuroregeneration in a child with cerebral palsy after cardiac arrest. The boy, whose cord blood was stored at birth, was 2.5 years old and normally developed when global ischemic brain damage occurred resulting in a persistent vegetative state. Nine weeks later, he received autologous cord blood (91.7 mL, cryopreserved, 5.75 × 10*e*8 mononuclear cells) intravenously. Active rehabilitation (physio- and ergotherapy) was provided daily, follow-up at 2, 5, 12, 24, 30, and 40 months. At 2-months follow-up the boy's motor control improved, spastic paresis was largely reduced, and eyesight was recovered, as did the electroencephalogram. He smiled when played with, was able to sit and to speak simple words. At 40 months, independent eating, walking in gait trainer, crawling, and moving from prone position to free sitting were possible, and there was significantly improved receptive and expressive speech competence (four-word sentences, 200 words). This remarkable functional neuroregeneration is difficult to explain by intense active rehabilitation alone and suggests that autologous cord blood transplantation may be an additional and causative treatment of pediatric cerebral palsy after brain damage.

## 1. Introduction

Brain injury from cardiac arrest in children results from global cerebral ischemia. The outcome varies with duration of resuscitation. Unfortunately, for those who survive cardiac arrest, brain damage may result in lifelong sequelae, for example, cerebral palsy, for which there is no causative cure at present [1]. Only recently experimental evidence has been produced showing that systemic transplantation of human mononuclear cord blood cells—known to contain stem cells—prevents the development of spastic paresis in a model of perinatal ischemia in rodents [2, 3]. These promising therapeutic effects led us to perform an autologous transplantation of cord blood mononuclear cells in a child with severe cerebral palsy on January 27th, 2009. A brief account of this report has been given elsewhere [4].

## 2. Material and Methods

### 2.1. Medical History

A 2.5-year-old boy was admitted to a municipal pediatric intensive care unit presenting unspecific gastrointestinal symptoms, persistent vomiting (40–50 times), and tachycardia (>200 bpm) after antiemetic treatment for three days. Past medical history was otherwise unremarkable, physical (walking at 16 months) and mental development, including speech competence (three-word-sentences) and cognition, were normal and appropriate for age. On the following day the boy's state deteriorated. There were bradycardia and hypotension, arterial oxygen saturation fell, consciousness was reduced (Glasgow Coma Scale score 8), and artificial ventilation just was intended, when cardiac arrest occurred (ventricular fibrillation). Immediate cardiopulmonary resuscitation began and the trachea was intubated, when a massive hemorrhage from the gastrointestinal tract required several osseous emergency blood transfusions. Atropine, three injections of epinephrine, and two defibrillations had no effect. After a fourth injection of epinephrine and Amiodarone (5 mg/kg body weight) infusion, only transient sinus rhythm occurred and a third defibrillation was necessary to re-establish circulation. Blood tests and temperature control (>39°C) revealed hypovolemic hyponatremic hypopotassemic septic shock with multiple organ failure (pH 7.05, BE 18.5 mmol/L, lactate 14 mmol/L, leukocytes >30,000/*μ*L, CRP 66 mg/L, GOT 5,540 U/L, GPT 2,420 U/L, CK 107,940 U/L, CK-MB massive increase, macrohematuria, and myoglobinuria) and disseminated intravascular coagulation. Resuscitation after cardiac arrest lasted >25 minutes. Thereafter, surgery discovered an ileus (volvulus, persistent omphaloenteric duct) that required removal of 90 centimeters necrotic Ileum. Primary artificial ventilation was continued for five days.

### 2.2. Neurologic Examination after the Insult

**Figure 1:**
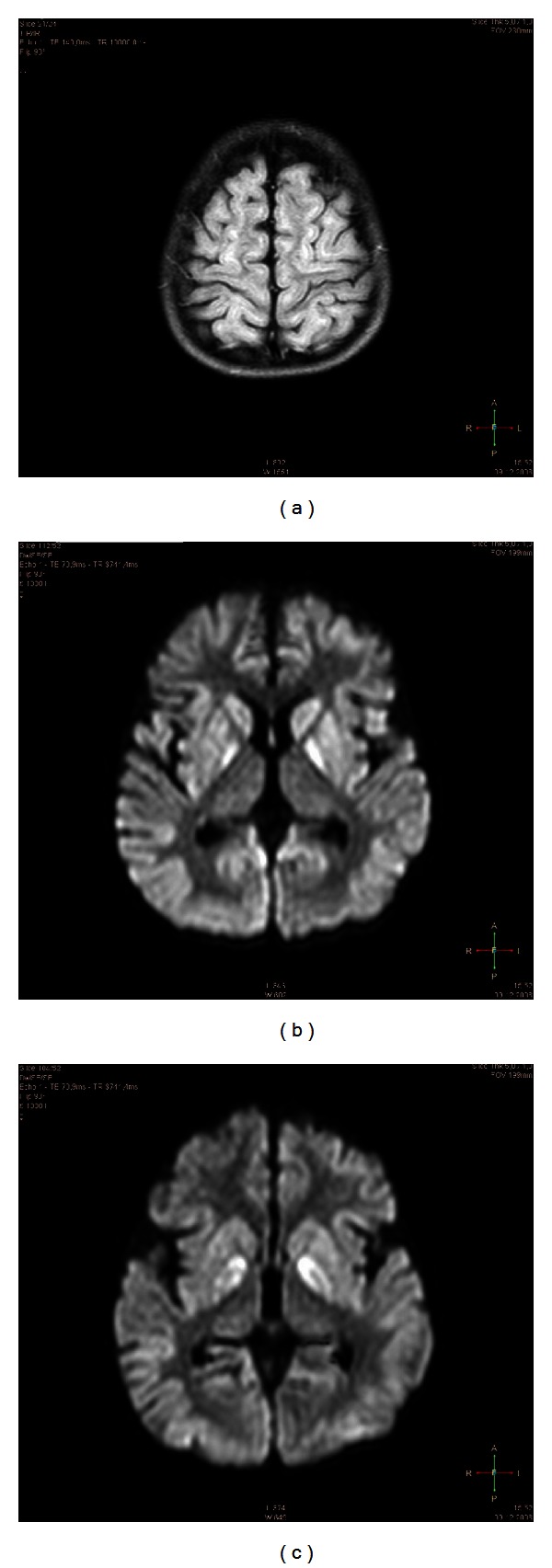
(a), (b), and (c) Brain MRI of the patient (L.B.) 2 weeks after cardiac arrest. Note signs of severe global ischemia in cortical structures as evidenced by (a) signal hyperintensity of gyri in almost entire cortex (FLAIR sequences) and basal ganglia (b), including caudate nucleus, pallidum, and putamen (c) (FLAIR DWI sequences with contrast media).

On extubation five days after cardiac arrest, spontaneous respiration occurred, but there was no motor activity and only minimal response to stimuli (Glasgow Coma Scale score 4). The pupils were dilated, showing minimal response to direct light. There were restlessness, smacking, decorticate posture, and continuous whimpering. Neuropediatric examination revealed global hypoxic-ischemic brain injury, confirmed by brain MRI (Figures 1(a), 1(b), and 1(c)) see video (S1, S2) in Supplemetary Material available online at http://dx.doi.org/10.1155/2013/951827. EEG rhythm and frequencies were grossly disturbed (<3/sec, poor amplitude <30 *μ*V, max. 100 *μ*V, transient isoelectric traces at one and two weeks). No side differences or signs of epilepsy were noted. There were generalized extensor spasticity, bilateral extensor spasticity in the lower, and flexor spasticity in the upper extremities. Therapeutic hypothermia was not provided. Three weeks after resuscitation the patient was referred to a rehabilitation center in a persistent vegetative state.

On admission at the rehabilitation center, a persistent vegetative state was confirmed. There were spontaneous eye opening, wide pupils, slow reaction to light, eye closure upon strong stimuli, right exophoria, positive corneal and oculocephalic reflexes, startle reaction on acoustic stimuli only, orofacial hypotonia, drooling, extensor spasticity, spasticity in both arms with elbow joint and wrist flexion, no motor control of the head, and hypotonia of the trunk. Hyperreflexia of the legs, positive Babinski signs on both sides, no spontaneous movements of the legs, continuous dyskinetic movements of the arms, hand-mouth contact, sluggish but preserved response to pain stimuli in sensomotor key areas, but no statomotor control were noted. The patient scored 0% in the first two dimensions “Lying & Rolling” and “Sitting” of Gross Motor Function Measure (GMFM). There were also profound sleep disturbance, absence of sleep-wake-cycles, severe restlessness, and absence of hearing and communication, including no visual contact or following of the eyes (cortical blindness). The patient cried and/or whimpered continuously. The initial score on the Coma Remission Scale was 8/24 [5]. Due to dysphagia, a stomach tube for enteral nutrition was used. 

### 2.3. Autologous Cord Blood Cell Transplantation

**Figure 2:**
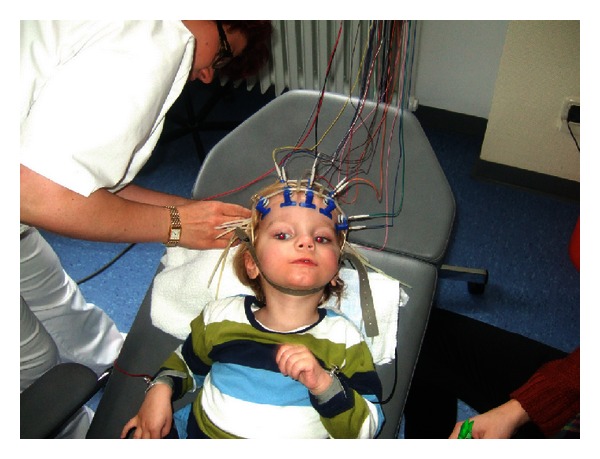
EEG recording (L.B.) before transplantation. The patient (L.B.) is in a persistent vegetative state 9 weeks after the insult before transplantation of cord blood cells. Note the dilated, unresponsive pupils in spite of bright light from the ceiling.

In this desperate situation the parents sought for alternatives and searched the literature as *ultima ratio *[3]. They contacted the Department of Obstetrics and Gynecology (Ruhr-University Bochum) to inquire about a potential individual treatment with their son's cord blood that had been collected at birth and stored in a blood bank (Vita34, Leipzig, Germany). After written informed consent of the local ethics committee and the parents was granted, autologous transplantation was prepared according to German legal requirements (AMG §41(2) [BGBl.1S.2631], guideline Bundesärztekammer). Identity cord blood unit/patient (CBU no. 10.03.98.82.1) was confirmed genetically (Gen.-no. PEI HG.00000.01.1/2). The neurologic examination before transplantation (video S3) included EEG (Figure 2) (10 : 20-system) and blood tests. The CBU contained 91.7ml blood, cryopreserved by 6.0% DMSO (w/v), with a total of 5.75 × 10*e*8 mononuclear cells without erythroblasts (vitality 85.7%, hemoglobin 125.7 mg/mL). After premedication (Midazolam), the unmanipulated CBU was transplanted intravenously over 40 minutes, nine weeks after cardiac arrest [6]. Moderate adverse effects (transient hemoglobinuria, nausea, and hypertension) were noted, and monitoring was continued for 36 hours. The patient was discharged to the rehabilitation unit, where physiotherapy, ergotherapy, and speech training were provided on a daily basis. Follow-up was at 2, 5, 12, 24, 30, and 40 months.

## 3. Results

**Figure 3:**
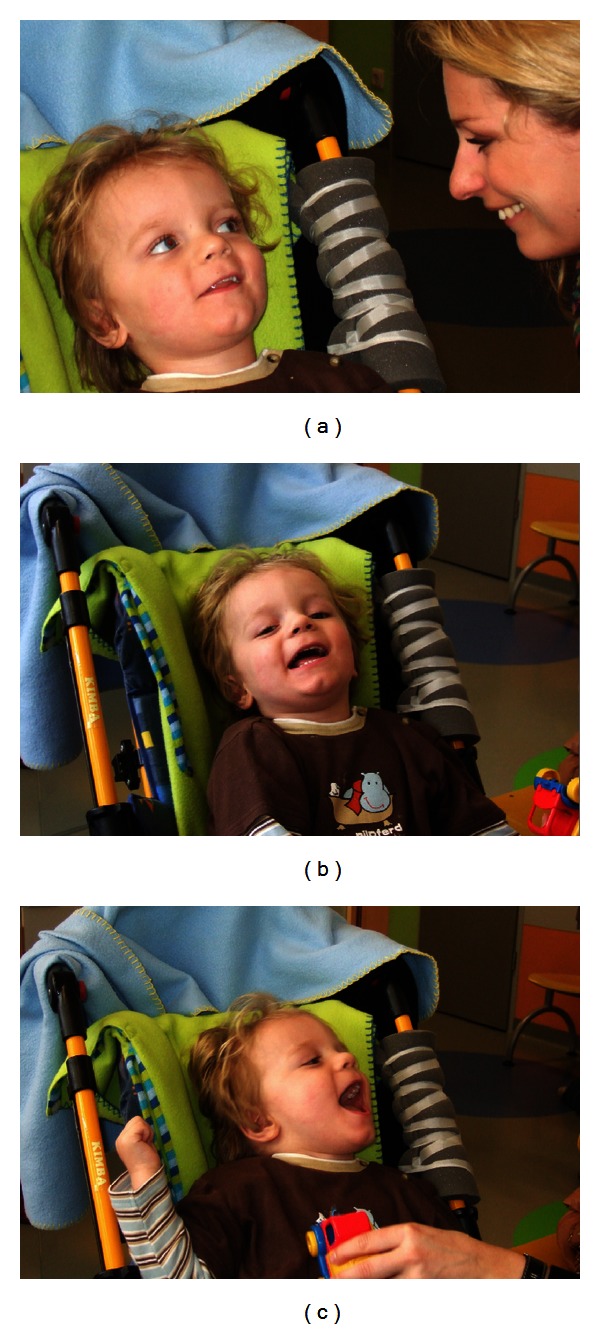
(a), (b), and (c) Two-months follow-up. (a) First social smiling of the patient (L.B.) towards his mother and (b, c) laughing, when played with, 2 months after autologous transplantation of cord blood cells (i.e., 4 months and one week after severe brain damage caused by cardiac arrest).

Following cord blood treatment, there were remarkable changes in psychomotor development. After one week, the patient stopped continuous whimpering and responded to acoustic stimuli. After four weeks, spasticity was slightly reduced and he could touch a “Big Mac” on command (poor fine motor control). After two months, motor control of the head improved, spastic paresis was reduced, and eyesight recovered in part, as did EEG. The patient could grasp, hold, bite, chew, and swallow a biscuit (video S4), developed social smiling (Figure 3(a)), laughed when played with (Figures 3(b) and 3(c)), and spoke the words “ma-ma”, apparently directed in part (video S4). On discharge, GMFM had improved to 23% from 0% (“Lying & Rolling” 37%, “Sitting” 8%) and Coma Remission Scale to 22/24 from 8. Neurologically, the patient showed a tetraparesis with emphasis on the legs. Sitting in a rehabilitation-buggy was possible, as was hand-mouth contact. However, the patient still presented a severe neurologic residual syndrome, including stereotypic hand-mouth exploration, hypotonic trunk, poor head control, central dyskinetic movement disorder in the arms, and persisting neonatal reflexes.

After five months, EEG was normal. The patient showed brief eye contact and fixing, followed objects, and was able to clap his hands, no tremor. He liked to play with toys appropriate for one-year-old, knew and demonstrated his organs (ear, nose, belly). His receptive speech competence and understanding was far better than the expressive.

After 1 year, the patient's social interaction, cognition, identification of animals, give-and-take, and fine motor control of both hands improved. He could grasp and eat peanut flips independently (movements of the hands slightly clumsy). Neurologically, he showed spastic tetraparesis with emphasis on legs. There were unsupported sitting, supported standing (balance problem), full motor control of the head, first independent rolling from prone to supine position, and first supported walking. 

After two years, there was independent eating and speech competence of eight words (pronunciation slurred, mimicking prosody) with broad understanding. The patient moved from a prone to a free sitting position and crawled without cross-pattern, but using the arms. Independent passive standing, walking with support, and independent locomotion in a gait trainer was possible (video S5). He played imaginative games, and recognized colours, animals, and objects, assigning them correctly. Fine motor control improved to such an extent that he managed to steer a remote control car (video S6). At 30 months, he formed two-word-sentences using 80 words.

After 40 months, there was further improvement in both receptive and expressive speech competence (four-word-sentences, 200 words), walking (Crocodile Retrowalker), crawling with cross-pattern, and getting into vertical position. 

## 4. Discussion

We report a remarkable neurologic functional regeneration after autologous transplantation of cord blood cells in a young boy who suffered from cerebral palsy and persistent vegetative state after cardiac arrest causing severe brain damage as evidenced by MRI.

Given the duration of resuscitation and the severity of the cerebral insult, the chances of the patient's survival were poor (6%), even though cardiac arrest occurred in-hospital [7]. In a recent study all children with cardiac arrest of >13 minutes who were not treated with ECMO died and mortality was positively associated with the number of dysfunctional organ systems, arrest duration >10 mins, the use of epinephrine, and the decision not to use ECMO as part of the arrest management [8]. In previous studies none of the patients who were given more than two doses of epinephrine or required resuscitation for more than 15 or 20 minutes survived to hospital discharge [9, 10]. In a prospective study on 129 in-hospital pediatric cardiopulmonary resuscitations, thirty-day survival decreased by 5% with each elapsed minute of resuscitation [11].

The neurologic outcome of survivors after global cerebral ischemia varies with duration of resuscitation [12, 13]. The positive predictive value for poor outcome, for example, severe disability, persistent vegetative state, or death, is 91% for duration of initial cardiopulmonary resuscitation exceeding 10 minutes and 100% for discontinuous EEG activity [12, 14]. Particularly poor is the prognosis, if children are comatose (Glasgow Coma Scale < 5), show absence of pupillary reflex at 24 hours, or lack the induced hypothermia as part of the arrest management [12, 15]. This is supported by MRI findings in the late subacute phase, that is, at 14–20 days [16]. None of the patients with cortical structure abnormalities recovered beyond a severely disabled state [17]. Thus, in our case the neurologic prognosis of the patient, who showed MRI signal hyperintensity in both cortex and basal ganglia, intermittent isoelectric EEG, and a persistent vegetative state when discharged from the hospital, was ominous if not hopeless. The long-term outcome of the latter condition in children is poor, about 40% die and, at best, children show only minimal awareness after an average of four and a half-year follow-up [18].

Though causality is impossible to establish, there is experimental evidence to support the view, that the functional neurologic regeneration observed may be in part mediated by “therapeutic” effects of the cord blood cells [19, 20]. As we have shown previously in a perinatal cerebral hypoxic-ischemia model in newborn rats [21], mononuclear cells derived from human cord blood show a highly specific “homing” and migrate into the lesioned region of the brain in large numbers within 24 hours when given intraperitoneally [2, 3]. This chemotactic process is mediated in part by stromal-derived factor (SDF)-1, a chemokine that is expressed in the lesioned brain, and transplanted human umbilical cord blood cells expressing the SDF-1 receptor CXCR4 migrate to the lesioned site [22]. Thus, neurotrophic (e.g., BDNF, VEGF), synaptotrophic (e.g., NGF), anti-inflammatory (e.g., Il-6,Il-8, Il-10), antigliotic (e.g., Cx43), antiapoptotic, and proangiogenic neuroregenerative effects are entrained resulting in significant functional neuroregeneration in that gross and fine motor function and coordination were restored [19, 23–27].

To our knowledge this is the first published scientific report on an individual treatment of cerebral palsy in humans by autologous transplantation of cord blood cells and hence a close look at the patient's neurologic development is warranted. It appears as if global hypoxia-ischemia of >25 minutes has in part reset neurologic development to that at birth. This is reflected by the neonatal reflexes observed and their disappearance over time. This holds also true for some milestones of neurologic development that were noted in the patient, for example, lifting (one month) and motor control of the head (four months), aimed reach (three months), free sitting (12 months), and refined pinch (12 months). In some cognitive categories the development of milestones was ahead of schedule, for example, hand-mouth exploration of objects (six months), give-and-take (12 months), pointing to known body parts (seven months), interest in illustrated children's books, and pointing to known objects (12 months), suggesting re-establishment of neuronal abilities present before the insult. For example, receptive speech competence and understanding was far better than the expressive, and first directed words (ma-ma, pa-pa) were spoken at seven months after the insult. Two-word sentences and expressive vocabulary of eight words were noted at two years, though pronunciation was somewhat slurred. At three-year follow-up, this further improved to four-word sentences using approximately 200 words expressive vocabulary accompanied by a remarkably broad understanding. Notably, speech competence improved beyond that before the insult. 

## 5. Conclusions

Thus, given the severity of brain damage and persistent vegetative state he suffered from, the patient has recovered to an extent, that is difficult to explain by intense active rehabilitation alone. Taking the evidence together, it appears that autologous transplantation of cord blood cells may in part have contributed to the remarkable functional neuroregeneration observed in this patient. If true, this would be the first account of a successful causative cell therapy of pediatric cerebral palsy, a condition for which there is no cure at present.

## Supplementary Material

Video S1: Brain MRI (FLAIR sequences). Brain MRI (FLAIR sequences) of a 2.5 years old patient (L.B.) 14 days after global hypoxic-ischemic insult caused by cardiac arrest. Note, massive damage as evidenced by signal hyperintensity in almost the entire cortex and basal ganglia.
Video S2: Brain MRI (FLAIR DWI sequences with contrast media). Brain MRI (FLAIR DWI sequences with contrast media) of a 2.5 years old patient (L.B.) 14 days after global hypoxic-ischemic insult caused by cardiac arrest. Note, massive damage as evidenced by signal hyperintensity in basal ganglia, caudate nucleus, putamen and particularly in the pallidum.
Video S3: Neurologic examination before transplantation. Neurologic examination of the patient (L.B.) in a persistent vegetative state before transplantation of cord blood cells. Note, severe cerebral palsy (tetraspastic ) 9 weeks after global hypoxic-ischemic insult caused by cardiac arrest. Note also, there is no motor control of the head.
Video S4: Functional neuroregeneration at two months follow-up. Functional neuroregeneration at two months follow-up after transplantation of cord blood cells. Note, the social smiling and response when the patient (L.B.) is addressed, independent eating of a biscuit, improved motor control of the head, recovered eyesight and hearing.
Video S5: Functional neuroregeneration at two years follow-up (a). Functional neuroregeneration at two years follow-up after transplantation of cord blood cells. The patient (L.B.) walks independently in a gait trainer, improved understanding and speaking in response to questions. Note, broad understanding of spoken context.
Video S6: Functional neuroregeneration at two years follow-up (b). Functional neuroregeneration at two years follow-up after transplantation of cord blood cells. The patient (L.B.) is steering a remote control car by console with much improved fine motor control in both hands and fingers. Note, the patient moves from prone to free sitting position.
